# Risks of Ear Complaints of Passengers and Drivers While Trains Are Passing Through Tunnels at High Speed: A Numerical Simulation and Experimental Study

**DOI:** 10.3390/ijerph16071283

**Published:** 2019-04-10

**Authors:** Pengpeng Xie, Yong Peng, Tiantian Wang, Honghao Zhang

**Affiliations:** 1 Key Laboratory of Traffic Safety on Tracks, Ministry of Education, School of Traffic & Transportation Engineering, Central South University, Changsha 410075, China; paulxie14@csu.edu.cn (P.X.); yong_peng@csu.edu.cn (Y.P.); zhanghh2016@csu.edu.cn (H.Z.); 2 Joint International Research Laboratory of Key Technology for Rail Traffic Safety, Central South University, Changsha 410075, China; 3 National & Local Joint Engineering Research Center of Safety Technology for Rail Vehicle, Central South University, Changsha 410075, China

**Keywords:** high-speed train, aural discomfort, human middle ear FE model, moving model tests, tunnel environment

## Abstract

Ear complaints induced by interior pressure transients are common experiences for passengers and crew members when high-speed trains are passing through tunnels. However, approaches to assessing the risks of the pressure-related aural discomfort have not been reported until recently. The objective of this study was to evaluate the hazards of interior pressure transients of high-speed train on human ears combining the effects of operation speed and seal index. Moving model tests were conducted to obtain the pressure transients when the model train runs in the tunnel. The recorded data were transformed into the interior pressures by empirical formula. Furthermore, the aural sensations were divided into four levels hierarchically and the range for each level was derived by logistic regression analysis method and represented by three biomechanical metrics. Furthermore, a human middle ear finite element (FE) model was used to simulate its dynamics under the interior pressures. The results indicate that lifting operation speed from 250 km/h to 350 km/h in tunnel will prolong the duration of ear complaints by more than two times whereas improving the seal index from 4 s to 12 s will reduce the incidences of the onset of tinnitus and hearing loss by more than ten times. In addition, the duration of aural comfort shortens from the head car to the tail car against the running direction. It is desirable that enhancing the seal index improve the aural sensations of the passengers and crew members considering the lifting operation speed of high-speed train.

## 1. Introduction

Due to the interactions between air, rail and train, a wide variety of concerns have emerged relating to aerodynamic noise, resistance, environmental and public health issues [1,2]. However, the interactions intensify when high-speed trains pass through a tunnel. This simultaneously gives rise to pressure fluctuations inside the train, which cause aural discomfort in the passengers and crew members [3]. In China, a large number of tunnels have been constructed ranging from hundreds of meters to tens of kilometers in length. For train drivers with long years of service in areas with numerous tunnels, the risks of suffering ear diseases are increased significantly [4]. Similarly, passengers travelling by the train undergo aural discomfort when the trains enter the tunnel. The aural sensations present with various symptoms such as aural fulness, otalgia, dizziness, temporal hearing loss and tinnitus, etc. [5,6]. Hence, the train-tunnel effect poses hazards to riding comfort and otological health.

Many efforts have been made to mitigate the negative consequences of interior pressure fluctuations on the passengers and crew members when a train passes through a tunnel. For example, Muñoz-Paniagua implemented a genetic algorithm to minimize the pressure gradient through optimization of the shape of the train nose [7]; the addition of different hood patterns at the tunnel entrance also alleviates the amplitudes of pressure gradients [8,9]. Matsubayashi et al. proposed an active control technique to downsize the pressure wave by emitting a wave which was superposed on the incident wave in the tunnel [10]. Despite the fact these findings highlighted that decreasing the pressure gradient and amplitude produced positive effects, it was not clearly elucidated how human ears respond to the ambient pressure fluctuations.

Ears, including the outer ear, the middle ear and the inner ear, are essential for humans to perceive ambient sounds and identify their direction. The middle ear is composed of the tympanic membrane (TM), the malleus, the incus, the stapes and the suspensory ligaments and tendons. It should be emphasized that the TM is responsible for converting the ambient pressure changes into mechanical vibrations and transmitting them to the inner ear by the ossicle chain path. The vibration transfers to the stapes footplate (SFP) until it is received by the inner ear. In healthy ears, large pressure fluctuations are certain to incur large vibration of both TM and SFP and in turn result in aural discomfort. It is obvious that the TM and the SFP play critical roles in whether and how much the pressure fluctuations are perceived by ears. Previous researches used human ear finite element models to investigate the middle ear functions as well as pathologies [11,12]. Kalb and Price have developed an auditory hazard assessment algorithm for humans (AHAAH) to predict the potential influences of impulsive noise on human ears [13,14]. However, frequency-independent pressure waves induced by train-tunnel effect are different from frequency-dependent noise or impulsive sounds and the hazards on ears caused by noise were generally judged by sound pressure level or the exposure duration [15,16]. Furthermore, the latent risks on human ears induced by the micro pressure waves generated by a high-speed train’s passing through tunnels has not been investigated until recently.

This study proposed a kind of biomechanical approach to predict the potential aural sensations that passengers and crew members may experience in a tunnel during travel by train. The core of this approach depends on two steps. One is to establish a uniform rule that correlates the characteristics of pressure fluctuation and the dynamics of human middle ear. The other is to collect the data of the interior pressure transients and to simulate the vibration of ear model under these pressure transients. Firstly, moving model aerodynamic tests were done on our lab’s aerodynamics platform by which the pressure transients in different car positions were collected. In sequence, the assessment standards were established and the recorded pressure data was exerted on the TM. By comparing the simulation results with the standards, assessment of the aural sensations was made throughout the trains’ travel in the tunnel. Furthermore, it sheds light on the evaluation of the attempts on improving the aerodynamic environments when the trains run or intersect in a tunnel.

## 2. Materials and Methods

### 2.1. Human Middle Ear Finite Element Model

**Figure 1:**
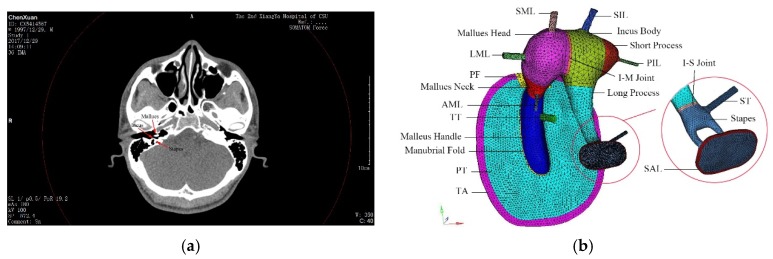
Human middle ear biomechanical finite element model; (**a**) ossicle bones on head computed tomography (CT) scanning; (**b**) ear model. Note: TA: tympanic annulus, PT: pars tensa, PF: pars flaccida, TT: tensor tympani, AML: anterior mallear ligament, LML: lateral mallear ligament, SML: superior mallear ligament, I-M joint: incudomalleolar joint, SIL: superior incudal ligament, PIL: posterior incudal ligament, ST: stapedial tendon, SAL: stapedial annular ligament.

Eight adult volunteers (seven males and one female, age: 21–31) were recruited and regular ear-nose-throat (ENT) medical examinations were completed before computed tomography (CT) scanning. This was done because it should ensure the individuals have no ear-related diseases that may affect the efficacy of the reconstructed ear model. Two volunteers were excluded due to otitis media and rhinitis diseases. By collecting the CT scanning data, one ear (right ear) of a male volunteer was selected for reconstruction. The human middle ear finite element model was reconstructed in accordance with CT scanning data. It was comprised of the TM, the malleus, the incus and the stapes bones, two joints and the suspensory ligaments as shown in Figure 1.

It is noteworthy that the auricle, the external air canal and the inner ear were eliminated because the outer ear is primarily responsible for sound collection and amplification while the inner ear is too complicated to simulate as a result of its functional mechanism [17]. Since the TM and the SFP are two dominant components and play key roles in energy transmission and conversion, such simplification would not downplay the results of the ear dynamics [12]. The model was constrained at the ring of the TM and the end faces of the suspensory ligaments and tendons except for the SAL [18]. With regard to the SAL, the nodes of its circumference were clamped. Besides, as a replacement of the inner ear, four dashpots were created at the periphery of the SFP [11]. Meanwhile, the pressure transients were loaded at the lateral side of the TM.

### 2.2. Division of Aural Discomfort Level

Japanese scientists have conducted airtightness experiments of the train cabin and revealed the correlations between the pressure changes and the aural discomfort [19]. The experiments were done in a range of no more than 3 kPa and 0.5 kPa/s. In this study, the four aural discomfort levels were hierarchically ordered as ideal, good, bad and worse. In detail, the ideal level represents when human ears hardly perceive the ambient pressure changes, the good level represents ears that sense the pressure changes but no ear complaints occur, whereas aural discomfort sensations start from the bad level and the ears become intolerant to such a change. The worse level is likely to happen if the pressure changes intensify in amplitude and gradient. According to the experimental data, a total of 116 pressure conditions were designed and exerted on the TM surface. There are 19 conditions for the ideal level, 20 for the good level, 38 for the bad level and 39 for the worse level.

What deserves attention is that three indicators were picked as the output variables and named as I1, I2 and I3 which respectively are the displacement of the TM umbo (the central position of TM), the displacement of the SFP and the velocity of the SFP. As the TM is the most sensitive organ of the human ears to barometric variations and closely related to the discomfort sensations like aural fulness and otalgia, I1 is much a competent predictor to evaluate these manifestations [20]. Moreover, the TM umbo is usually considered as a fundamental reference point to present the dynamics of the middle ear [21]. On the other hand, the SFP is connected to the oval window of the inner ear, the oscillation of the middle ear excites the vibration of the inner ear as well. Thus, the pressure-related vertigo or vomiting are thought to be correlated with the large values of I2 [22]. Furthermore, I3 is a key determinant for the generation of the pressure gradient between the oval window and the round window [23,24]. The pressure gradient in turn triggers bioelectrical signal in the cochlear which was transduced to the brain. As a result, the aural sensations were perceived and identified. Thus, I3 is a good indicator to assess the inner-ear-related aural discomfort.

The responses of the three indicators were then analyzed by the logistic regression method. A binary coding approach was used to obtain the risks of one aural discomfort level under certain exposures. For example, the results derived from the ideal level were coded as 0 while those from the good level were coded as 1. Based on the outcomes of logistic regression, S-shaped regression curves can be generated according to function in Equation (1) [25]:

(1) p(x)=1(1+e(α−βx))

where *α* is the intercept and *β* is the regression coefficients of exposures of the indicators. The parameters were determined by maximum likelihood method to maximize the function’s fit to the data. Likewise, the same procedure was used to obtain the regression curves between other levels.

### 2.3. Moving Model Aerodynamic Tests

**Figure 2:**
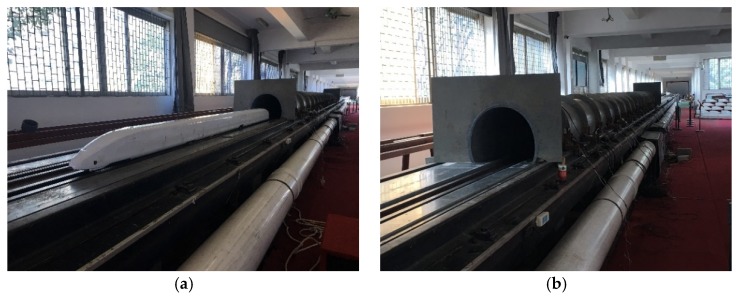
Moving model tests of aerodynamics in tunnels. (**a**) model train; (**b**) model tunnel.

Moving model tests of trains passing through a tunnel were carried out at the Central South University’s moving model test platform in China, for the application of the numerical method introduced in this manuscript. The test platform has obtained China Metrology Accreditation (CMA) qualification (certificate number 2014002479K). More detailed information about the platform can be found in [26,27]. A certain type of three car marshalling train (79.77 m in length) and a 70 m^2^ standard single-car tunnel (350 m in length) are adopted in the test, as is shown in Figure 2. All the models are scaled down to 1:20 in the test. The operating speeds are 250 km/h, 300 km/h and 350 km/h, respectively. Each case is repeated 12 times, whose acceptability in terms of train speed is examined. Tests that do not meet the accuracy of ±0.3% of the target train speed are discarded.

The aerodynamic effects caused by a real train passing through a tunnel can be effectively simulated using scaled models and moving model experimental devices if a similarity criterion is satisfied. The Mach number, blockage ratio, scaling of the train and tunnel geometry, and the Reynolds number are the main similarity parameters needed to be considered for the similarity criterion [27,28,29,30,31]. The first three parameters of the moving model tests in this manuscript are agree to the similarity criterion. The Reynolds number in this study is from 9.69 × 10^5^ (corresponding to 250 km/h) to 1.36 × 10^6^ (corresponding to 350 km/h) and that of the real train is from 1.94 × 10^7^ to 2.71 × 10^7^. According to the EN Standard 14067–4 [32], when the Reynolds number of the scaled model test is larger than 2.5 × 10^5^, the aerodynamics coefficients are nearly invariable with increasing Reynolds numbers [27,30,31,33]. As a result, the results of moving model tests of simulating a real train passing through a tunnel is reliable and the aerodynamics coefficients of moving model tests can represent that of the real train.

**Figure 3:**
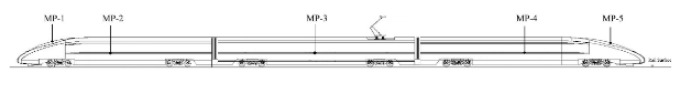
Distribution of measurement points.

The positions of measurement points are set as shown in Figure 3. A DC030NDC4 pressure sensor (Honeywell, Morris Town, NJ, USA) was selected for the pressure measurements. The sampling frequency of sensors, in this set of tests, is 1 kHz. Before each test, every sensor must be recalibrated to the normal function of the sensors. The errors of the loading test are within 0.2%, which is considered to meet the test accuracy requirements. The average values of the pressure transients of the measurement points on the train surface (numbered as MP-1, MP-2, MP-3, MP-4, MP-5) are selected for study, which is defined as P_out_.

Pressure transients inside train can be transformed from pressure transients outside train based on the empirical equation in reference [34]. The equation can be written as:

(2) $$\omega =\frac{P_{out}-P_{in}}{dP_{in}/dt}$$

where *ω* in the equation represents dynamic seal index, which is set as 4 s, 8 s and 12 s in this paper. *P_out_* and *P_in_* represent the exterior and interior pressure transients, respectively.

The *ω* is obtained by pressure tests. According to the Chinese high-speed train standard, the train internal pressure is increased to 4000 Pa (relative pressure) and naturally relieves the pressure to 1000 Pa without any other influence. The value of the period of time of the process is defined as the dynamic seal index *ω*. Due to the high running speed, the process of high-speed trains that have been in service for several years will last about 4 s ~ 12 s. As a result, the *ω* is set as 4 s, 8 s and 12 s for this study.

## 3. Results

### 3.1. Risks Analysis of Aural Discomfort

**Figure 4:**
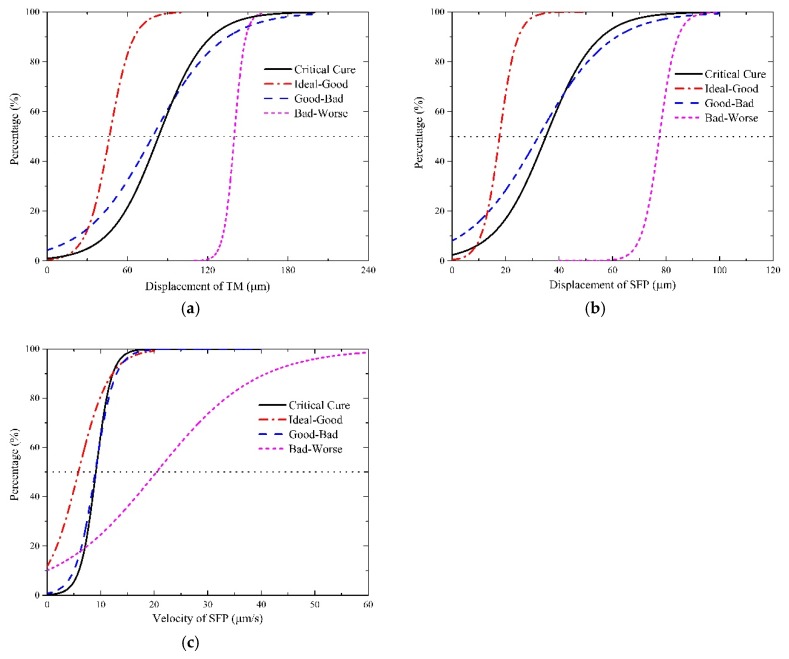
Relationship between risks of the aural discomfort level and the exposures of the indicators. (**a**) I1; (**b**) I2; (**c**) I3.

Table 1 listed the outcomes of the analysis and Figure 4 shows the incidences of each level under certain exposures. Before presenting the results, some specifications should be made. Four denotations were used to represent the threshold values between the aural discomfort levels, which are T1, T2 and T3, respectively. T1 was the threshold between the ideal level and the good level, T2 was that between the good level and the bad level, T3 was that between the bad level and the worse level. Meanwhile, T4 was used as the critical threshold between the aural comfort and the aural discomfort.

Table 1 presented that the outcomes of the regression analysis are statistically significant because all the p-values are under 0.05. Figure 4 revealed the incidences of the onset of one aural discomfort level for given values of the indicators. It indicated that the curves shift to the right side when discomfort level deteriorates from the ideal to the worse. Also, the good-bad curve is very similar to the critical curve in the three illustrations. The thresholds between two levels were confirmed when the incidence of each level accounts for fifty percent. As shown in Figure 4, a dotted horizontal line was plotted in each illustration. The abscissa values of the intersection points between the horizontal line and the S-shaped curves were the expectation values of the thresholds. Table 2 lists the threshold values for each indicator. It should be noted that Tc was introduced as the average of T2 and T4.

Virtually, both T2 and T4 can represent the boundary between the aural comfort and the aural discomfort. It is noteworthy that the values of T2 were approximately the same as those of T4. Moreover, the relative errors between them were less than 5%. The deviation between T2 and T4 was attributed to the bias of sample sizes, because the coefficients of the critical curve was determined by 116 sample points whereas the good-bad curve was generated by 58 points. Therefore, in this study, the average value of T2 and T4, Tc, was used to discriminate the aural comfort from the aural discomfort. To sum up, the intervals of all the discomfort levels were (0, T1), (T1, Tc), (Tc, T3), (T3, >T3), respectively. When assessing the aural sensations induced by the interior pressure changes, the first step was to obtain the responses of the three indicators and the next step was to decide in which interval the responses lies. The intervals for each aural discomfort level were tabulated in Table 3 as below.

### 3.2. Pressure Distributions in Train Cabins

**Figure 5:**
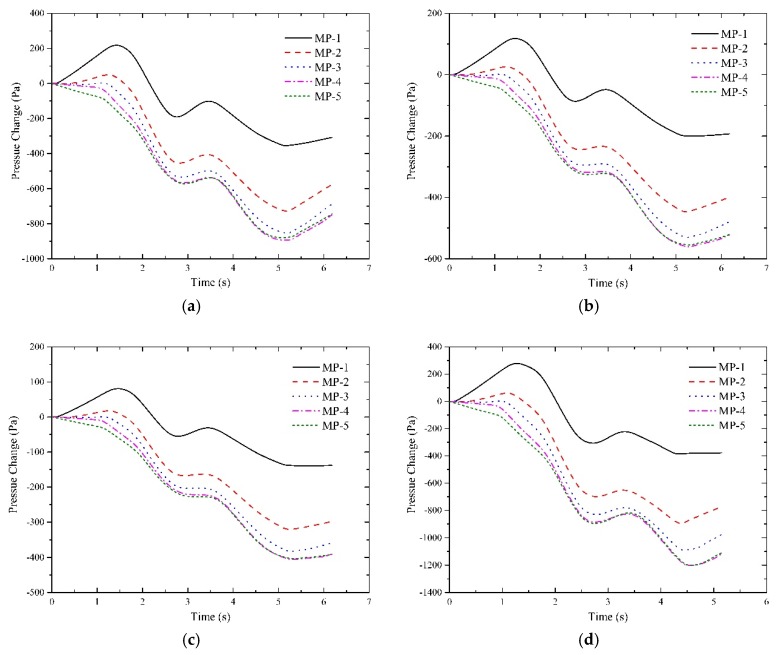

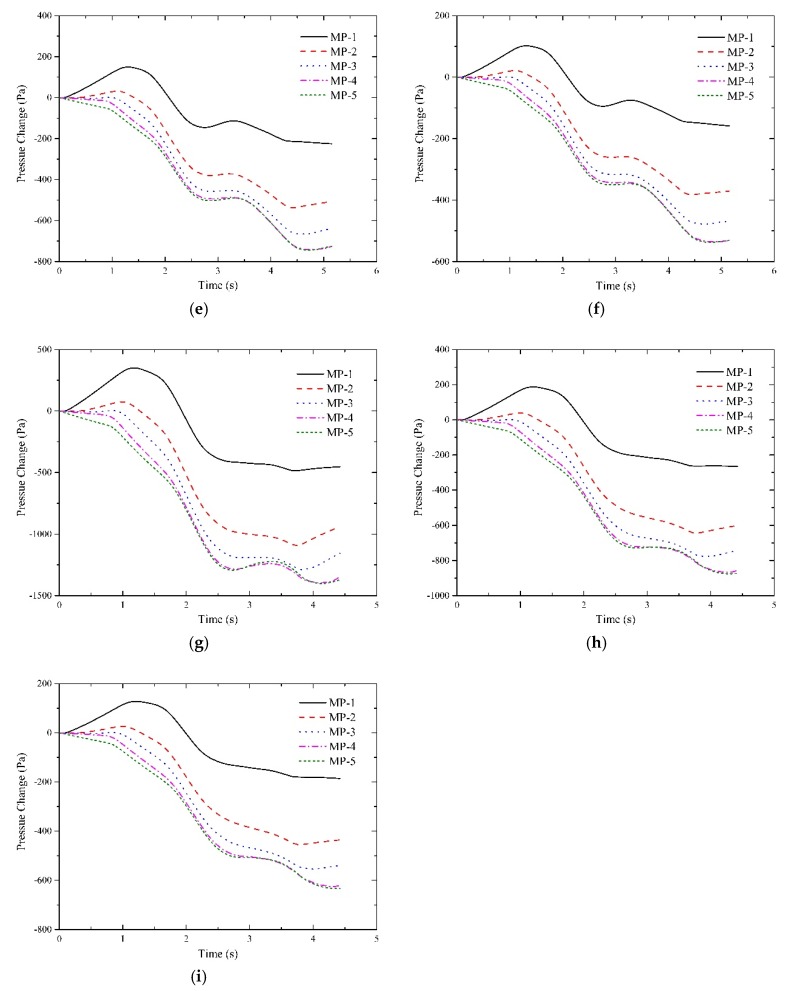
The interior pressure changes at different operation speed and seal indexes of train body. (**a**) S-4 at 250 km/h; (**b**) S-8 at 250 km/h; (**c**) S-12 at 250 km/h; (**d**) S-4 at 300 km/h; (**e**) S-8 at 300 km/h; (**f**) S-12 at 300 km/h, (**g**) S-4 at 350 km/h; (**h**) S-8 at 350 km/h; (**i**) S-12 at 350 km/h.

The interior pressure transients of the tested cabins at three operation speed levels were displayed in Figure 5.

It should be explained that S-4, S-8, S-12 denote that the seal indexes of the train body were 4 s, 8 s and 12 s, respectively. As illustrated in Figure 5, the characteristics of the pressure transients in all the tested cabins were consistent. The interior pressure increases from zero in the head car from the instant that the train enters the tunnel. After reaching the peak, it begins to fall from positive to negative until the tail car departs from the tunnel. By comparison, it is observed that the positive peak value in the driver cabin is larger than that in the passenger cabin with respect to the head car. Whereas in other tested cabins, the likelihood that the positive pressure occurs in cabins decreases from the middle car to the tail car. Table 3 gives an overall comparison of the maximum pressure differences in the cabins.

Table 4 reveals that the pressure difference increases against the moving direction of the train model from the driver cabin of the head car to that of the tail car at a given operation speed. Also, it increases with the lift of operation speed for all the tested cabins. However, it decreases when the seal index increases. In Discussion section, how the pressure changes affect travelers’ and crew members’ aural sensations will be presented.

### 3.3. Middle Ear Vibration under Pressure Transients

**Figure 6:**
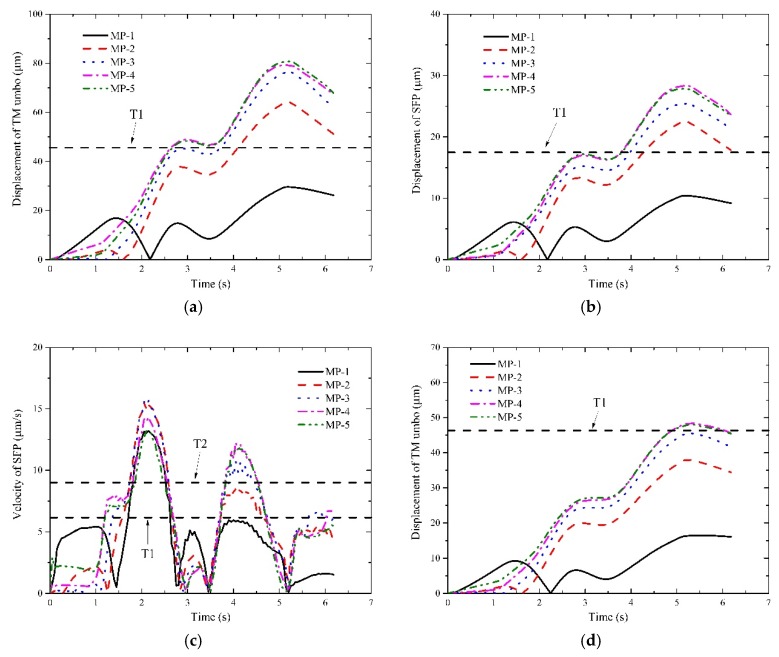

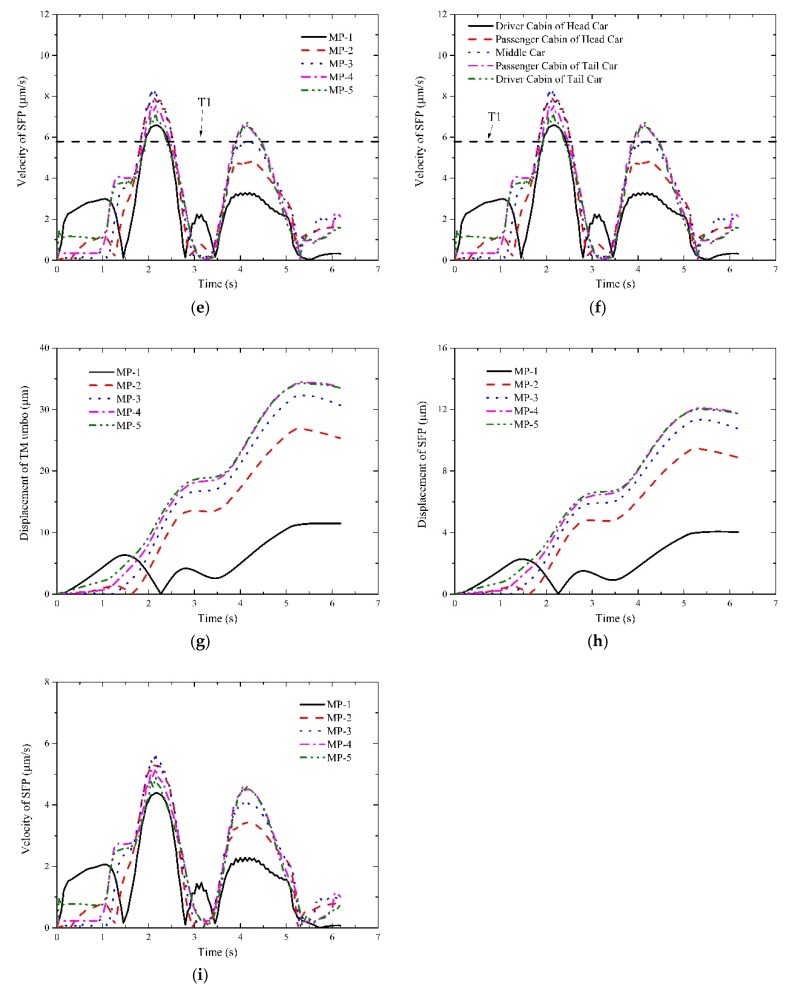
Responses of the indicators of interest at 250 km/h. (**a**) responses of I1 under a seal index of 4 s; (**b**) responses of I2 under a seal index of 4 s; (**c**) responses of I3 under a seal index of 4 s; (**d**) responses of I1 under a seal index of 8 s; (**e**) responses of I2 under a seal index of 8 s; (**f**) responses of I3 under a seal index of 8 s; (**g**) responses of I1 under a seal index of 12 s; (**h**) responses of I2 under a seal index of 12 s; (**i**) responses of I3 under a seal index of 12 s.

**Figure 7:**
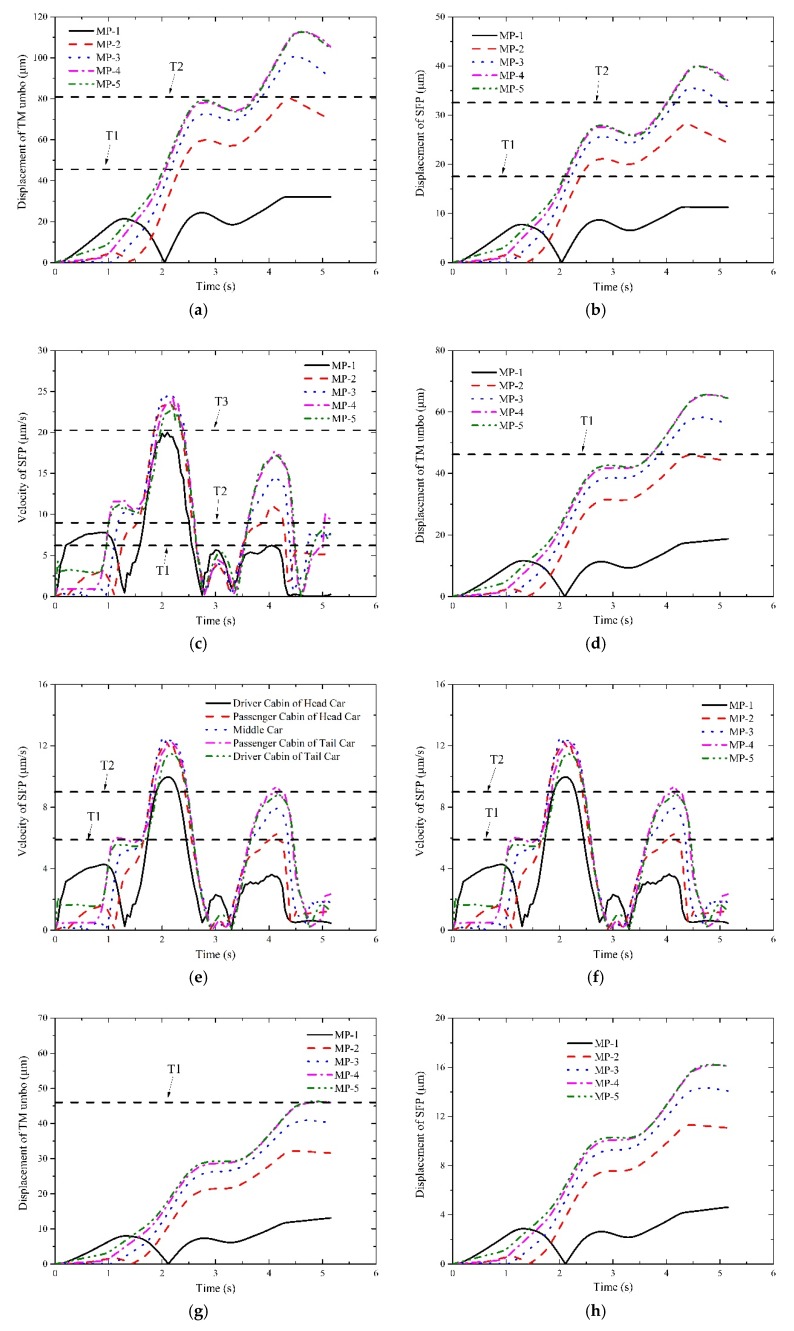

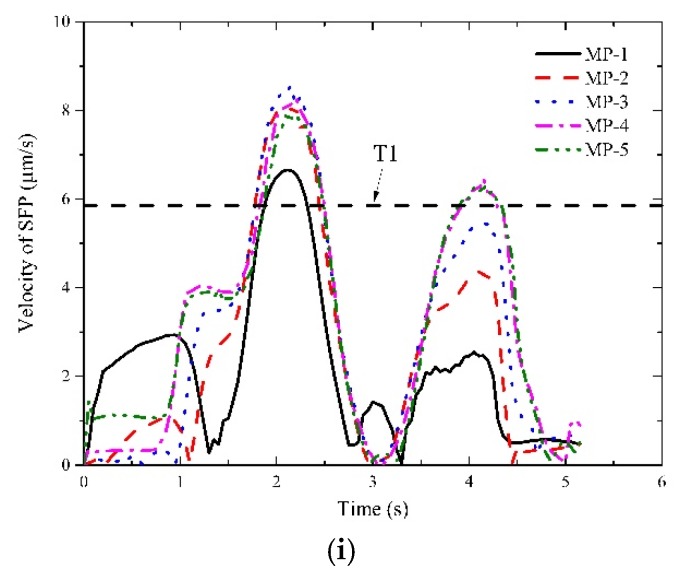
Responses of the indicators of interest at 300 km/h. (**a**) responses of I1 under a seal index of 4 s; (**b**) responses of I2 under a seal index of 4 s; (**c**) responses of I3 under a seal index of 4 s; (**d**) responses of I1 under a seal index of 8 s; (**e**) responses of I2 under a seal index of 8 s; (**f**) responses of I3 under a seal index of 8 s; (**g**) responses of I1 under a seal index of 12 s; (**h**) responses of I2 under a seal index of 12 s; (**i**) responses of I3 under a seal index of 12 s.

**Figure 8:**
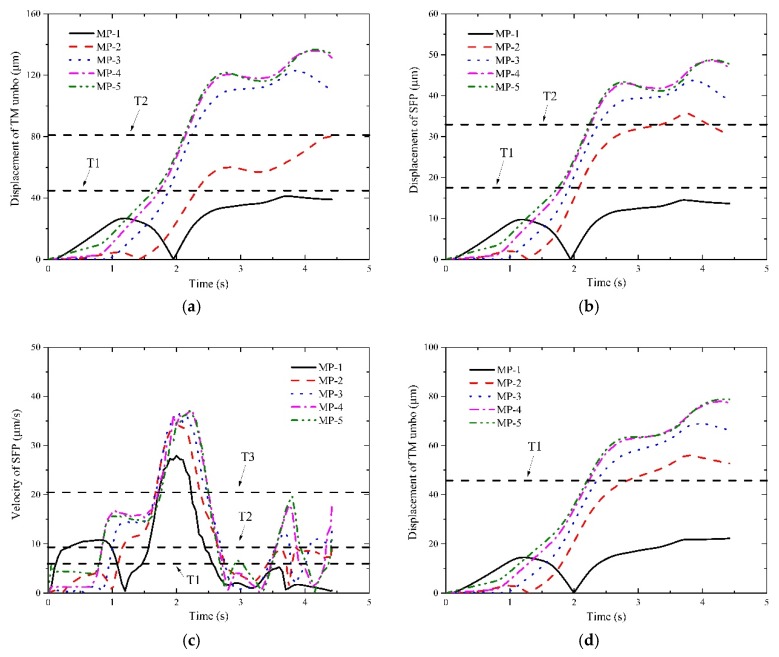

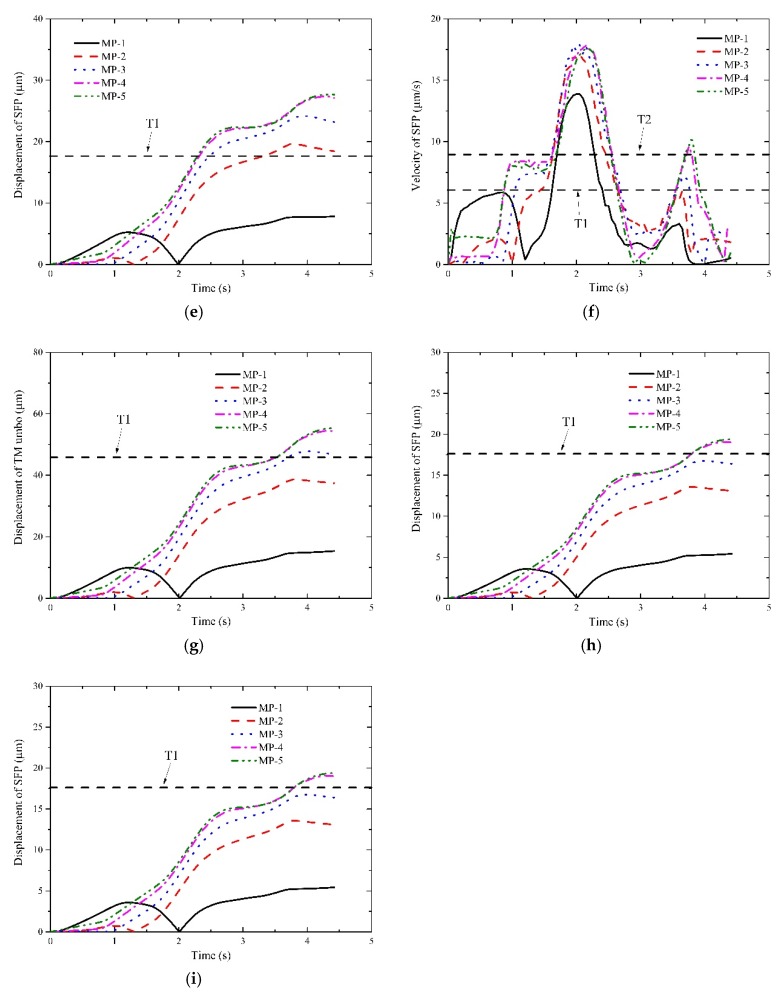
Responses of the indicators of interest at 350 km/h. (**a**) responses of I1 under a seal index of 4 s; (**b**) responses of I2 under a seal index of 4 s; (**c**) responses of I3 under a seal index of 4 s; (**d**) responses of I1 under a seal index of 8 s; (**e**) responses of I2 under a seal index of 8 s; (**f**) responses of I3 under a seal index of 8 s; (**g**) responses of I1 under a seal index of 12 s; (**h**) responses of I2 under a seal index of 12 s; (**i**) responses of I3 under a seal index of 12 s.

The dynamic responses of middle ear throughout the train’s passing through tunnels are displayed in Figure 6, Figure 7 and Figure 8 at different operation speeds.

It deserves attention that the horizontal dashed lines were plotted in some illustrations for reference purposes to judge which levels the interior pressure transient may affect. In detail, if no lines are plotted, it indicates the aural feelings stay within the ideal level; if one or more lines are plotted, they are the threshold values of the ideal level, the good level and the bad level from the bottom to the top, respectively. It also implies the aural feelings transform into the good level from the ideal level and remain within good level till the train departs. If only one reference line is plotted, it implies that the aural sensations stay in the good level when the response curves overpass the line or T1 line. Likewise, if two or three reference lines are plotted, it means the aural sensations of the bad level even the worse level are perceived.

With respect to I1 and I2, their response curves of MP-1 point and MP-2 point ascend at the beginning, fall to zero after reaching the peaks and ensue constantly increase till the tail car leaves the tunnel. Whereas for other measurement points, the curves increase continuously until the train approaches the tunnel exit. The reason for the difference between them is that the two points experience a significant phase of positive pressure initially and a relatively long phase of negative pressure. Thus, the TM moves inward first and slowly recovers to the undisturbed state with the decrease of the positive pressure. When the negative pressure appears in both points, the TM begins to deform outward till the end. The movement of TM will certainly involve the SFP to move similarly.

With respect to I3, the curves of MP-1 point and MP-2 point are also different from those of others. For both points, their curves begin with a sharp increase first and stabilize for less than 1 s, then fall steeply nearly to zero. During the remained time, the curves experience two or three peaks and decrease after the last peak till the train leaves the tunnel. While for other points, two increase phases and a stable phase are displayed before the first peaks appear. During the following time, the curves of all the measurement points exhibit consistency. As are displayed in Figure 6, Figure 7 and Figure 8, the aural discomfort sensations mainly outburst when the train is running approximately in the middle of the tunnel. Furthermore, high seal index or degraded operation speed is beneficial to reduce the likelihood of the onset of the aural discomfort. In Section 4, how these factors influence humans’ aural feelings will be discussed in detail.

## 4. Discussion

In this section, three factors which respectively are the operation speed of the train, seal index and positions of measurement points were considered. Furthermore, their effects on the onset of aural discomfort were investigated using the control variable method. For example, I2 and I3 were regarded as invariables when the effect of I1 was to be investigated.

Here, some explanations should be elucidated with respect to how the three indicators mentioned above correlate with the aural discomfort sensations. As high pressure amplitude causes large deformation of the TM, a variety of barotraumas may occur such as ear pain, bleeding or perforation [6,35,36,37]. Armstrong and Heim found that the TM bulges and sensation of aural fulness and pains occurs when the negative pressure in the external ear canal reaches 1.6 kPa. Moreover, tinnitus starts, usually accompanied by vertigo with benign nausea, if the negative pressure continues decreasing [38]. Hence, it is sufficient to believe that large displacement value of TM or I1 is doomed to incur such aural complaints as otalgia, fulness and even bleed or rupture. The SFP is connected to the oval window of the inner ear which is composed of vestibular semi-duct and cochlea, the vibration input from the oscillation of the TM is transmitted to the inner ear and in turn elicits movement of both vestibular semi-duct and cochlea. If the SFP vibrates with a high magnitude, the semi-duct will be affected which may induce vertigo or vomit sensations [22]. On the other hand, the vibration of the SFP excites the oval window to vibrate and in turn the flow of perilymph in the scala tympani [23]. The key process for triggering bioelectrical signal to the brain is to excite the flow of fluid between scala tympani and scala media under the pressure gradient [24]. As the impedance is the pressure at per unit areas and per unit velocity, a pressure gradient can be produced by the effect of the velocity of the SFP. Driven by the pressure gradient, the hair bundles on the basilar membrane vibrate and thereby bioelectrical was generated and transferred to the brain. It is obvious that the velocity of the SFP or I3 is a key factor to judge the sensations of inner ear. It is widely recognized that noise exposure potentially results in tinnitus symptoms for those involved with loud sound environments [39,40,41]. As was reported by Aibara et al., the velocity of the SFP reaches more than 10 μm/s at a sound stimulus of 90–120 dB which is sufficient to elicit slight tinnitus [12]. It therefore implies that the real value that triggers the onset of tinnitus is virtually lower than 10 μm/s. As is indicated in Table 2, the simulated threshold values of I3 is 9.04 μm/s. Therefore, large velocity of the SFP is interpreted to cause ear complaints like tinnitus and hearing loss.

In the following sections, the effects of operation speed, seal index and car number on the three indicators will be discussed separately. Furthermore, statistical analysis is completed to figure out the duration time of each level for each condition according to the results as were shown in Figure 6, Figure 7 and Figure 8. Then the effect is quantified by calculating the percentage that each level accounts for relative to the total time. This helps us interpret how and what kinds of the aural discomfort sensations emerge under the interior pressure changes.

### 4.1. Effect of Operation Speed on Aural Discomfort

**Figure 9:**
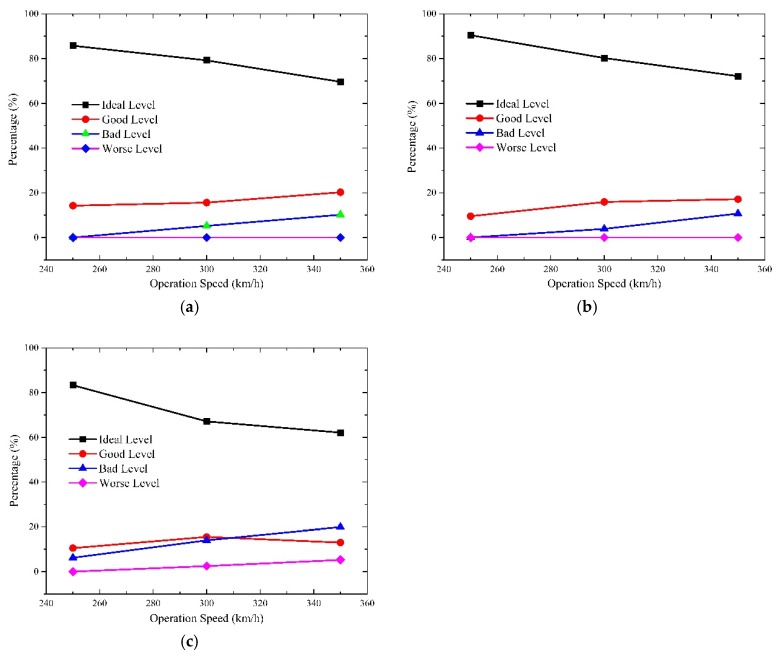
The relationship between operation speed and aural discomfort; (**a**) I1; (**b**) I2; (**c**) I3.

To investigate the effect of the operation speed, the other two factors are treated as invariables. Thus, the total time of every discomfort level at each operation speed is calculated by adding the duration time of each measurement point under different seal indexes. Figure 9 displays the characteristics of the aural discomfort at different speeds.

Figure 9 clearly indicates that the percentages of the ideal level of the indicators descend while that of bad level increases with the increase of operation speed. With respect to that of the good level, I1 and I2 increases slightly while I3 goes up first and then drops when the operation speed accelerates. The percentages of I1 and I2 at the worse level stabilize at zero level with the increasing operation speed, while the proportion of I3 at the worse level climbs from zero at 250 km/h to 5% at 350 km/h.

The results reveal that the duration of aural comfort of ideal level reduces when the operation speed of train accelerates. Because the duration of aural discomfort can be calculated by adding the percentage of the bad level and that of the worse level, the likelihood of aural discomfort at 350 km/h increases more than two times than that at 250 km/h for the three indicators. With respect to the manifestations of aural discomfort sensations, the incidence of episodes of the aural fulness, otalgia and vertigo increase by two or three times when the operation speed lifts from 250 km/h to 350 km/h. Besides, it is four times more likely to induce the onset of tinnitus and temporal hearing loss at 350 km/h compared with 250 km/h.

Sanok et al. revealed that the more severe the pressure fluctuations the more likely the increase in the level of the aural discomfort perceived by subjects in pressure chamber tests [42]. As is indicated in Figure 5, higher operation speed gives rise to more severe pressure fluctuations. It is therefore more likely to induce exacerbating aural discomfort. Sato et al. disclosed that aural discomfort is associated with amplitudes and gradients of pressure transients and risks of aural discomfort become higher if speed elevated [43]. As was illustrated in Figure 5, higher speed results in higher amplitudes of negative pressure in cabins. Thus, it implies that lifting the operation speed of a train in the tunnel is much more likely to cause symptoms of aural discomfort like tinnitus and hearing loss.

### 4.2. Effect of Seal Index on Aural Discomfort

**Figure 10:**
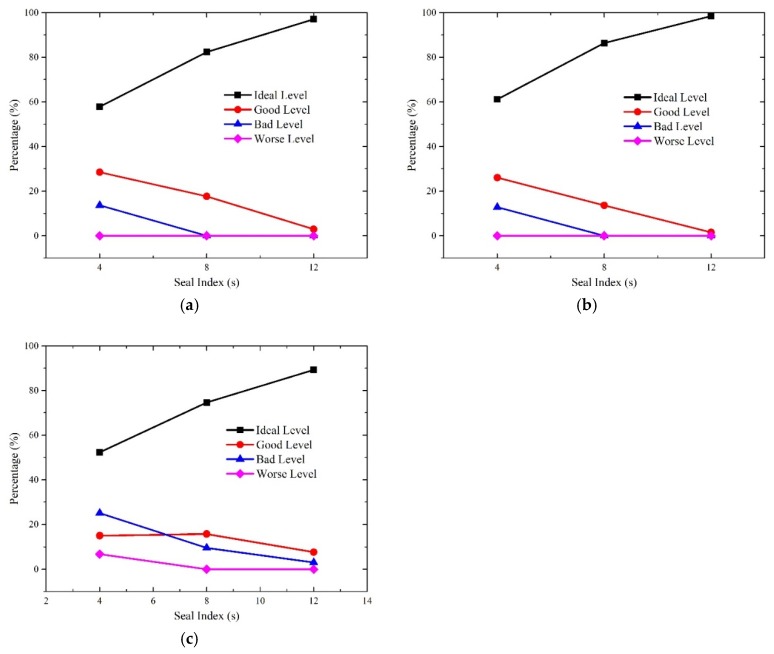
The relationship between seal index and aural discomfort. (**a**) I1; (**b**) I2; (**c**) I3.

By using the control variable method, the effect of seal index on the aural discomfort was analyzed as shown in Figure 10.

As is illustrated in Figure 10, the percentage of the ideal level of the indicators increases significantly when the seal index increases, whereas that of the other levels decreases more or less. It can be found that the proportion of the ideal level accounts for nearly more than 90% when the seal index is 12 s. That is, the duration of aural discomfort at the ideal level lasts longer when the seal index becomes larger. The probability of ear annoyance like otalgia or vertigo falls to zero once the coefficient reaches 8 s. Moreover, the likelihood of the onset of tinnitus also reduces to a very low level but not zero with a coefficient of 12 s. Adding the percentages of the bad level and the worse level, it indicates that the duration of aural discomfort like fulness and otalgia falls from 13.7% to zero and the duration of vertigo dropped from 12.8% to zero when the seal index varies from 4 s to 12 s. For the duration of tinnitus or hearing loss, it reduced significantly from 31.9% to 3% as the seal index increases from 4 s to 12 s. Generally, the aural comfort sensations tend to sustain longer when the seal index rises. Figure 5 displays that increasing the seal index of the train body leads to descending pressure amplitudes and thereby yielded improved aural sensations [44,45]. Thus, it is transparent that increasing the seal index of the train body is beneficial to improve passengers’ and crew members’ aural experiences.

### 4.3. Effect of Car Number on Aural Discomfort

**Figure 11:**
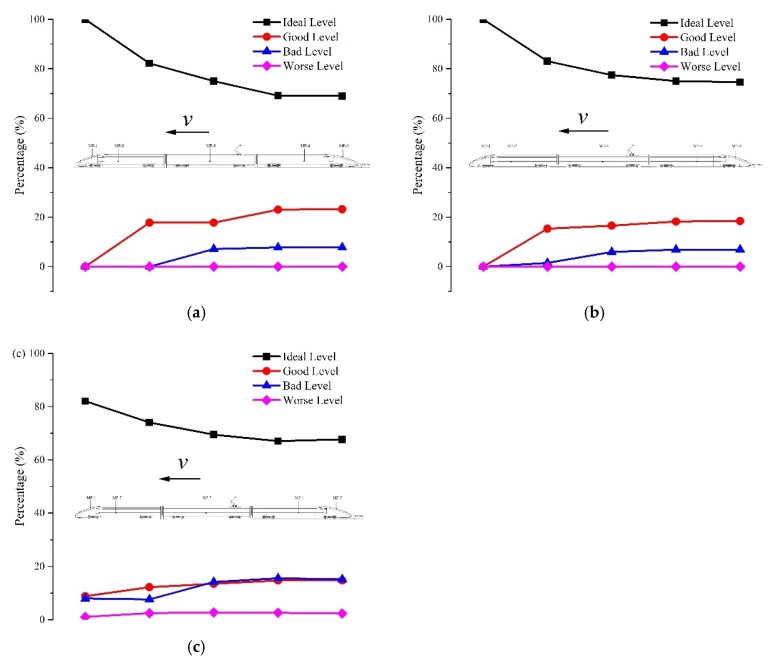
The relationship between car number and aural discomfort. (**a**) I1; (**b**) I2; (**c**) I3.

To compare the aural feelings of passengers and crew members in different cabins, Figure 11 was generated to illustrate the differences in aural sensations against the running direction of the train.

In Figure 11, there are five points plotted in each curve which correspond with the five measurement points. They are the driver cabin of the head car, the passenger cabin of the head car, the cabin of the middle car, the passenger cabin of the tail car and driver cabin of the tail car respectively from the left side to right side. The results show that the duration of the aural comfort at the ideal level goes down while the lasting time at the good and the bad levels rises from driver cabin of head car to that of tail car. Besides, the time at the worse level for I3 in Figure 11c increases slightly but no significant changes are observed in Figure 11a,b. As a whole, the duration of the aural comfort sensations accounts for more than 80% in all the cabins. The onset incidence of tinnitus and hearing loss is larger than that of the aural fulness and vertigo. Furthermore, the incidence rises from the driver cabin of the head car to that of tail car. Thus, it is conclusive that the aural sensations of humans on board deteriorate gradually against the running direction of the train.

## 5. Conclusions

This study attempted to predict the aural discomfort experienced by the passengers and crew members when a train is passing through a tunnel. Moving train model tests were completed to obtain the pressure variations on the outer surface of the train body at three different operation speeds. By empirical formula that builds up the relationship between the pressure transient outside the train and that inside the train, the pressure change histories of five measurement points of interest were figured out. On the other hand, a kind of the aural discomfort assessment methodology was established based on the findings of the cabin airtightness experiments which revealed the relationship between pressure changes and the onset of tinnitus. The pressure loads in experiments were exerted on the lateral side of TM and the responses of the human middle ear FE model were produced. Besides, four discomfort levels were divided hierarchically from ideal, good, bad to worse according to the results of experiment data. Three indicators: the displacement of the TM (I1), the displacement of the SFP (I2) and the velocity of the SFP (I3) were employed. Logistic regression analysis method was implemented to determine the thresholds between adjacent levels. Furthermore, the correlations between the indicators and the ear complaints were elucidated.

The results reveal that the thresholds between adjacent discomfort levels from the ideal to the worse respectively were 46.61 μm, 80.81 μm, 139.87 μm for I1, 17.76 μm, 32.97 μm, 77.51 μm for I2 and 5.84 μm/s, 9.04 μm/s, 20.45 μm/s for I3. The assessment results of the aural discomfort highlighted that the aural annoyance begins when the train is running in the middle of the tunnel. Also, the incidences of the onset of tinnitus or hearing loss is higher than that of aural fulness, otalgia and vertigo. With respect to the effects of the pressure transients on the aural sensations in different cabins, it is found that the duration of the aural comfort shortens from the driver cabin of the head car to that of the tail car whereas the incidence of the aural discomfort rises. Moreover, the results highlight that enhanced airtightness of train body and degraded operation speed is beneficial to improve the aural experiences of passengers and crew members when the train passes through tunnel.

When high-speed trains pass through tunnels, the potential hazards on human ears are due to two components. One is the pressure transients inside the train and the other is the amplified sound pressure level. In this study, our work was focused on the assessment of pressure-related aural discomfort without considering the effect of noise. Therefore, the aural discomfort level may be underestimated. In the future research, we will evaluate the aural discomfort by combination of both pressure-related and noise-induced discomfort.

## Figures and Tables

**Table 1:** Outcomes of logistic regression analysis for the three indicators.

	T1			T2			T3			T4		
	*α*	*β*	*p*	*α*	*β*	*p*	*α*	*β*	*p*	*α*	*β*	*p*
I1	5.504	0.118	0.002	3.092	0.039	0.001	34.5	0.254	0.000	4.637	0.056	0.000
I2	5.553	0.313	0.004	2.433	0.075	0.001	24.385	0.315	0.001	3.710	0.110	0.000
I3	2.017	0.345	0.025	4.938	0.550	0.000	2.190	0.107	0.000	6.311	0.693	0.000

**Table 2:** Threshold values for all aural discomfort levels.

Indicators	Threshold Values				
	T1	T2	T3	T4	T_c_
I1: μm	46.61	78.87	139.87	82.74	80.81
I2: μm	17.76	32.31	77.51	33.63	32.97
I3: μm/s	5.84	8.98	20.45	9.10	9.04

**Table 3:** Interval of each discomfort level.

Indicators	Aural Discomfort Level			
	Ideal	Good	Bad	Worse
I1: μm	(0, 46.61)	(46.61, 80.81)	(80.81, 139.87)	>139.87
I2: μm	(0, 17.76)	(0, 32.97)	(32.97, 77.51)	>77.51
I3: μm/s	(0, 5.84)	(0, 9.04)	(9.04, 20.45)	>20.45

**Table 4:** The pressure differences in different cabins (Unit: Pa).

Measurement Point	Operation Speed								
	250 km/h			300 km/h			350 km/h		
	Seal Index								
	S-4	S-8	S-12	S-4	S-8	S-12	S-4	S-8	S-12
MP-1	572	318	220	662	375	261	837	453	314
MP-2	779	473	339	955	569	404	1168	684	481
MP-3	856	531	383	1092	667	480	1290	777	555
MP-4	894	561	406	1201	742	536	1396	868	626
MP-5	888	556	404	1201	745	538	1401	875	634
